# Surgical strategies to avoid Glenn physiology during staged left heart recruitment

**DOI:** 10.1016/j.xjon.2026.101924

**Published:** 2026-06-12

**Authors:** Gianna Dafflisio, Arjun Verma, Aditya Suyakumar, Michael Bamgbose, Yasuyuki Kobayashi, Rebecca Beroukhim, Sunil Ghelani, Eric Feins, Sitaram M. Emani

**Affiliations:** a Department of Cardiac Surgery, Boston Children's Hospital, Harvard Medical School, Boston, Mass; b Department of Cardiology, Boston Children's Hospital, Harvard Medical School, Boston, Mass

**Keywords:** biventricular repair, borderline ventricle, ventricular recruitment

## Abstract

**Objectives:**

Staged left ventricular recruitment can promote left ventricular growth through controlled volume loading. The optimal pulmonary blood flow source and the need for the bidirectional Glenn during staged left ventricular recruitment remain unclear. This study investigates staged left ventricular recruitment outcomes in patients who underwent staged left ventricular recruitment with or without the bidirectional Glenn.

**Methods:**

This is a single-institution retrospective review of children undergoing staged left ventricular recruitment (2014-2024). Patients were stratified by pulmonary blood flow source: nonbidirectional Glenn (shunt/Sano upsize or pulmonary artery band alone) and bidirectional Glenn.

**Results:**

Eighty-eight patients underwent staged left ventricular recruitment with shunt upsizing (n = 11), pulmonary artery band (n = 9), or bidirectional Glenn/super-Glenn (n = 68). Median age at recruitment was 231 days (interquartile range, 495.25). Fundamental diagnosis was hypoplastic left heart syndrome/complex in 44 patients (50%). Median duration of recruitment was 642 days (576.5); duration was significantly lower in the nonbidirectional Glenn group compared with the bidirectional Glenn group (321 [480] days vs 754 [481] days) (*P* = .02). Forty-five patients (51.1%) achieved biventricular conversion, and 17 patients (19.3%) achieved 1.5-ventricle circulation. For the nonbidirectional Glenn, 90% reached biventricular circulation. Left ventricle indexed end-diastolic volume significantly increased during recruitment for the nonbidirectional Glenn (31.7 [28.6] mL/m^2^ to 57.7 [34.5]) (*P* < .001) and bidirectional Glenn (22.7 [14.3] mL/m^2^ to 48.5 [29.4]) (*P* < .001). Superior vena cava pressure significantly increased in the bidirectional Glenn group (9.5 [6] mm Hg to 16.5 [5] mm Hg) (*P* < .001) but not in the nonbidirectional Glenn group. Lymphatic complications occurred in 16% of patients and venovenous collaterals in 65% of patients in the bidirectional Glenn group. No patients in the nonbidirectional Glenn group experienced these problems.

**Conclusions:**

Shunt upsizing and pulmonary artery band modification can be effective for ventricular volume loading during staged left ventricular recruitment, enabling biventricular conversion while avoiding potential complications of bidirectional Glenn physiology. These strategies should be considered in patients not anticipated to require prolonged recruitment and in patients deemed poor bidirectional Glenn candidates.

## Graphical Abstract

**Figure:**
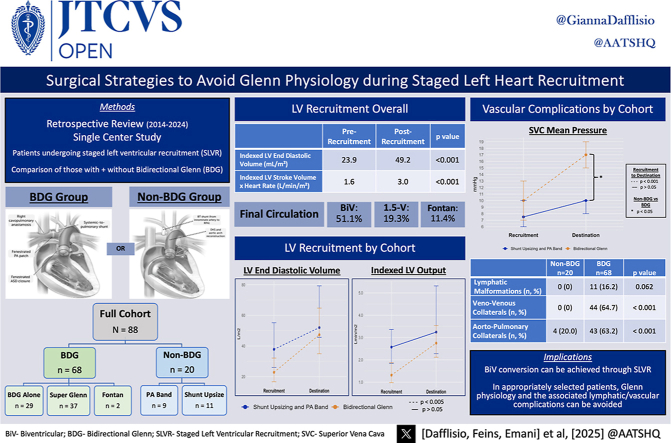

**Figure:**
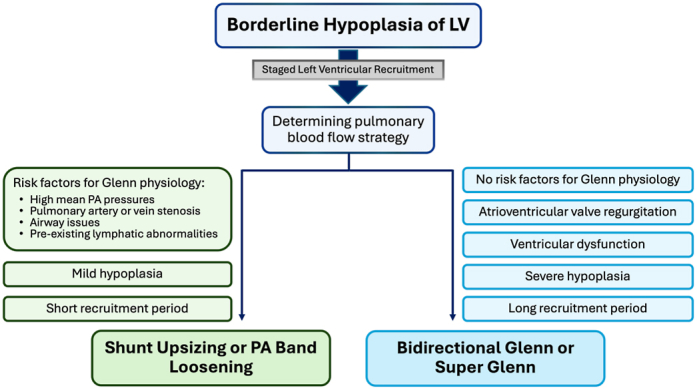
Pulmonary blood flow strategy for staged LV recruitment of borderline LV.

**Central Message**

Successful ventricular loading and BiV conversion can be achieved through staged ventricular recruitment while avoiding Glenn physiology and the associated lymphatic/vascular sequelae.

**Perspective**

The optimal pulmonary blood flow source for SLVR remains unclear. We evaluated ventricular growth in patients receiving SLVR with and without the Glenn. Both groups demonstrated successful ventricular recruitment and BiV conversion. Avoidance of the Glenn resulted in fewer lymphatic/vascular complications. Ventricular loading for SLVR can be done without Glenn physiology.

Surgical management for patients with a hypoplastic left heart is often decided in infancy. Patients with milder disease may be good candidates for primary biventricular (BiV) repair, whereas patients with severe left heart hypoplasia undergo single ventricle palliation (SVP). Although SVP has improved significantly over the years, the long-term complications and rates of Fontan failure remain problematic.^1^, ^2^, ^3^, ^4^ For this reason, there have been efforts to develop surgical strategies to perform initial SVP with ventricular recruitment and ultimately BiV conversion. This strategy is termed “staged left ventricular recruitment” (SLVR). SLVR promotes growth of the hypoplastic left ventricle (LV) and valvular structures through staged volume loading.^5^ Many prior studies have demonstrated success in increasing indexed ventricular size and output through SLVR.^6^, ^7^, ^8^

There are several options for pulmonary blood flow and ventricular volume loading at the time of SLVR. The “super-Glenn” consists of superior cavopulmonary connection (bidirectional Glenn [BDG]) with the addition of a systemic to pulmonary shunt (ie, aortopulmonary shunt or right ventricle [RV] to pulmonary artery [PA] conduit). Volume loading with a super-Glenn leads to growth of the hypoplastic LV and BiV and 1.5-ventricle conversion.^9^ However, the BDG or super-Glenn at the time of SLVR predisposes the patient to elevated pressure in the superior vena cava (SVC) and increases the risk of SVC syndrome, venovenous collateral formation, and lymphatic complications.

Volume loading with an aortopulmonary shunt or RV-PA conduit as the sole source of pulmonary blood flow at the time of SLVR may avoid disadvantages of the BDG. There are a small number of reports that have described shunt upsizing without creating a BDG as an effective recruitment strategy to achieve BiV circulation in patients with less severe hypoplasia.^6^^,^^10^, ^11^, ^12^ However, it is unclear whether the source of pulmonary blood flow at the time of SLVR impacts clinical outcomes or ventricular growth. The present study sought to evaluate outcomes in patients undergoing SLVR and compare outcomes between patients undergoing shunt upsizing, PAB, or BDG at the time of SLVR.

## Materials and Methods

### Study Cohort

Boston Children's Hospital maintains a prospectively collected database of all patients undergoing or being evaluated for ventricular recruitment toward BiV repair. This database is stored on the institutional REDCap server (Institutional Review Board No. P00043051, approval July 27, 2022). The BiV database was queried for all patients who underwent or are undergoing left heart recruitment at our institution and had their initial palliation surgery between 2014 and 2024. From this query, patients were excluded if they did not have at least 1 recruitment procedure done at our institution. Patients were separated into 2 cohorts. The non-BDG cohort was composed of patients who never underwent a BDG before, or as part of, their left heart recruitment. The BDG cohort was composed of patients whose left heart recruitment included a Glenn or super-Glenn.

### Data Collection

Patient demographics, anatomic diagnoses, preoperative imaging, operative details, and postoperative outcomes were collected from a REDCap database query and primary patient chart review. Postoperative outcomes included hospital length of stay, lymphatic complications (eg, chylothorax, plastic bronchitis, and protein losing enteropathy), vascular complications (eg, venovenous collateral formation, aortopulmonary collateral formation, and interventions for collateral closure), reinterventions after destination circulation, transplant, and mortality.

### Recruitment Patient Selection

A patient may be considered to have borderline left heart due to LV hypoplasia or left-sided valvular hypoplasia with a z-score between −2 and −5. Endocardial fibroelastosis and systolic or diastolic LV dysfunction may or may not be present in patients with borderline left heart. Patients with variants of hypoplastic left heart syndrome (HLHS) including aortic or mitral valve (MV) atresia were not considered candidates for SLVR. All other patients with borderline LVs were offered SLVR at our center, especially patients with concomitant risk factors for Fontan circulation.

### Recruitment Parameters and Definitions

For the BDG cohort, the start of recruitment was defined as the time of fenestrated atrial septation. For the non-BDG cohort, the start of recruitment was defined as the time of shunt/Sano upsizing or pulmonary artery band (PAB) loosening. Additional recruitment procedures included atrioventricular valve repair, aortic valve repair, LV endocardial fibroelastosis resection, and resection of LV outflow tract obstruction. For the non-BDG cohort, Sano and Blalock-Taussig-Thomas shunts were typically upsized by 1 to 1.5 mm. Decision-making regarding shunt type (ie, Sano vs Blalock-Taussig-Thomas shunt) was made on a case-by-case basis. Recorded recruitment parameters included initial (prerecruitment) palliation, operative details, interstage interventions, and destination circulation. “Baseline” was defined as immediately before the initial recruitment procedure.

### Imaging Analysis

Patient anatomic, volume, output, and pressure measurements were collected from imaging reports at 2 timepoints per patient: before initial recruitment (prerecruitment) and before establishment of destination circulation (predestination). Cardiac magnetic resonance (CMR) imaging was used preferentially for volume and output data, and transthoracic echocardiography was used for anatomic data/z-scores. If no CMR was available, cardiac computed tomography was used. Imaging studies were included if done within 1 year preceding the target surgery. If more than 1 CMR report was available in the year prior, the report within closest proximity to the upcoming surgery was chosen. Pressure and output measurements were collected from cardiac catheterization reports. Volume and output measurements included LV ejection fraction, LV mass:volume ratio, LV end-diastolic volume index (LVEDVi), indexed LV stroke volume (LVSVi), indexed LV output (defined as LVSVi × heart rate), and indexed MV inflow. Anatomic measurements included aortic valve annulus diameter, aortic root (AoR) diameter (anterior-posterior direction), MV annulus diameter (lateral direction), and associated z-scores. Hemodynamic measurements included LV end-diastolic pressure (LVEDP), cardiac index (calculated by Fick's principle), mean left atrial pressure, mean PA pressures, mean SVC pressure, LV outflow tract gradient, and atrial septal defect gradient.

### Statistical Analysis

Categorical variables were reported using frequency/percentage and analyzed using the Pearson chi-square test or Fisher exact test if the expected number was below 5. Continuous variables were summarized using median and interquartile range. When analyzing continuous variables across timepoints, the Wilcoxen rank-sum test was used for nonparametric variables. When analyzing the change in continuous variables across cohorts, mean change scores were compared using the Mann–Whitney *U* test. To account for multiple-comparison error, *P* values were adjusted using the Bonferroni correction. Data analysis was completed on R 4.5.0 (R Development Core Team).

## Results

### Patient Characteristics

A total of 88 patients met inclusion criteria for SLVR at our institution with initial palliation between January 2014 and July 2024. The non-BDG cohort (shunt/Sano upsizing or PAB modification alone) comprised 22.7% of the study population (n = 20). The BDG cohort (including super-Glenn) comprised 77.3% of the study population (n = 68). Within the non-BDG cohort, 11 patients (12.5%) had shunt alone: Three patients underwent stage I (Damus-Kaye-Stansel [DKS]) with Sano, 3 patients underwent stage I with Blalock-Taussig-Thomas shunt, 2 patients underwent a Sano without DKS, and 3 patients had a Blalock-Taussig-Thomas shunt without DKS. An additional 9 patients (10.2%) received PAB alone. In the BDG group, 29 patients (33.0%) underwent BDG and 37 patients (42.0%) underwent the super-Glenn at the time of SLVR, with 4 of the BDG patients later undergoing the super-Glenn as part of staged palliation. Two patients (2.3%) had Fontan circulation at SLVR.

Of the whole cohort, 35 patients were female (39.8%), the median age at time of initial palliation was 7 days (interquartile range, 13), age at initial recruitment procedure was 231 days (495.25), and age at establishment of destination circulation (ie, BiV conversion) was 2.85 years (2.27). Fundamental diagnosis was HLHS (or hypoplastic left heart variant) in 44 patients (50.0%), atrioventricular canal defect (AVC) in 25 patients (28.4%), double-outlet RV without AVC in 16 patients (18.2%), and transposition of the great arteries without AVC in 2 patients (2.3%). When comparing demographic and diagnostic characteristics between non-BDG and BDG cohorts, there was a significant difference in gender, age at initial palliation, age at destination, body surface area at destination, diagnoses, and initial palliation (Table 1). Before SLVR, LVEDVi, LVSVi, and mean PA pressure were higher in non-BDG compared with BDG patients.

**Table 1:** Patient characteristics

Variable	Non-BDG cohort n = 20	BDG cohort n = 68	*P* value
Gender (female: n, %)	12 (60.0)	23 (33.8)	.07
Age (d: median, IQR)			
At initial palliation	12 (39)	6 (10.25)	.02
Start of recruitment	222 (180)	264 (538.25)	.10
Destination	670.5 (652.25)	1062 (587.25)	.003
BSA (median, SD)			
Start of recruitment	0.39 (0.10)	0.37 (0.18)	.64
Destination	0.48 (0.17)	0.59 (0.15)	.01
Diagnoses (n, %)			.001
HLHS and variants	7	37	
AVC	10	15	
Balanced	1	1	
Right dominant	9	14	
Double-outlet right ventricle without AVC	0	16	
Transposition of the great arteries without AVC	2	0	
Down syndrome	1	2	
Initial palliation (n, %)			<.001
Stage 1 (DKS) with Sano or BTT	6	55	
Sano/BTT shunt without DKS	5	0	
Hybrid stage 1	0	1	
PA band alone	9	12	
Anatomic/hemodynamic metrics (median, IQR)			
Indexed LV end-diastolic volume (mL/m^2^)	37.8 (29.2)	22.8 (15.5)	.0133
Indexed LV stroke volume (mL/m^2^)	23.7 (15.4)	12.6 (9.2)	.0062
Mean PA pressure (mm Hg)	17.0 (5.3)	14.0 (3.50)	.0351

*BDG*, Bidirectional Glenn; *IQR*, interquartile range; *BSA*, body surface area; *HLHS*, hypoplastic left heart syndrome; *SD*, standard deviation; *AVC*, atrioventricular canal defect; *DKS*, Damus-Kaye-Stansel; *BTT*, Blalock-Taussig-Thomas; *PA*, pulmonary artery; *LV*, left ventricle.

### Recruitment and Operative Details

Median time from recruitment to destination for the overall cohort was 642 days (576.5). The non-BDG cohort had a significantly shorter time from recruitment to destination compared with BDG (321 days [480] vs 754 days [481]) (*P* = .02). Hospital length of stay after initial recruitment surgery for the full cohort was 14.4 days (24.3), with no significant difference between the non-BDG cohort (27.3 days [30.0]) and BDG cohort (14.5 days [19.6]) (*P* = .183). Hospital length of stay after destination surgery for the full cohort was 13.7 days (21.2), with no significant difference between non-BDG (14.2 days [34.6]) and BDG cohorts (13.2 days [14.5]) (*P* = .757).

Of the 88 patients in the study, 16 (18.2%) did not, or have yet to, make it to a destination circulation. Of these 16 patients, 12 are currently in recruitment (13.7%), 3 died (3.4%), and 1 (1.1%) is currently awaiting heart transplant due to diastolic dysfunction. Of the 3 patients who died, 1 had recurrence of severe pulmonary vein stenosis with concurrent sepsis, 1 had systolic and diastolic heart failure with concurrent sepsis, and 1 had multiorgan failure secondary to macrophage activation syndrome and interstitial lung disease. Of the 72 patients who reached a destination circulation, 44 (50.0%) reached a BiV repair with systemic LV, 1 (1.1%) had a BiV repair with systemic RV (via reverse double switch operation), 5 (5.7%) had a 1.5-ventricle repair with systemic LV and Glenn, 12 (13.6%) had a reverse 1.5-ventricle repair with systemic RV and Glenn, and 10 (11.4%) progressed to Fontan. In non-BDG patients, 18 of 20 patients (90%) underwent full BiV conversion with a systemic LV, whereas in BDG patients, 31 of 68 patients (45.6%) underwent BiV/1.5 conversion with a systemic LV (*P* < .002). Of these 31 BDG patients, 26 (38.2%) achieved a full BiV repair, and 5 patients (7.4%) underwent a 1.5-ventricle repair. BDG patients with HLHS type anatomy were less likely to achieve BiV conversion (8/37, 21.6%) than those with non-HLHS diagnoses (18/31, 58.1%) (*P* < .01). For 61.4% of patients (n = 54), the initial SLVR procedure was the only recruitment surgery before reaching destination therapy. Twenty patients had 2 recruitment surgeries, but no patient had more than 2.

Of the 68 patients in the BDG cohort, 29 patients (42.6%) had their BDG performed at a different institution. When comparing BDG patients who had their BDG performed elsewhere versus at our institution, patients at our institution were significantly smaller at the time of BDG (body surface area of 0.34 [0.04] vs 0.55 [0.14], *P* < .001). Patients at our institution also had significantly worse LVEDVi (17.6 [12.9] vs 28.0 [17.9], *P* < .002), LVSVi (10.3 [6.0] vs 15.9 [12.4], *P* < .004), and cardiac index (3.5 [1.2] vs 4.4 [1.4], *P* < .04).

### Impact of Staged Ventricular Recruitment on Left Heart Dimensions and Pressures

Anatomic, volume, output, and pressure variables were compared from prerecruitment to predestination imaging for all patients with HLHS-type anatomy. MV annulus z-score significantly improved from −2.3 (2.2) to −2.1 (3.5) (*P* = .017), and AoR diameter z-score improved from −1.1 (1.3) to 0.9 (2.2), although the increase was not significant. Ventricular volumes were significantly greater at destination compared with prerecruitment. LVEDVi increased from 23.9 mL/m^2^ (18.6) to 49.2 mL/m^2^ (32.3) (*P* < .001), LVSVi increased from 14.6 mL/m^2^ (11.6) to 29.7 mL/m^2^ (19.6) (*P* < .001), and indexed LV output (defined as LVSVi × heart rate) increased from 1.6 L/min/m^2^ (1.3) to 3.0 L/min/m^2^ (1.6) (*P* < .001). LVEDP also increased from 9.5 mm Hg (4.0) to 10.5 mm Hg (4.0) (*P* = .012), as did mean PA pressure (15.1 mm Hg [standard deviation: 3.9] to 18.8 mm Hg [6.0]) (*P* < .001) and mean SVC pressure (9.4 mm Hg [3.1] to 15.4 mm Hg [4.5]) (*P* < .001).

Within the non-BDG cohort, there were no statistically significant changes in MV annulus z-score from prerecruitment to destination (−2.0 [–2.3] vs −1.4 [3.0]) or AoR diameter z-score (0.6 [2.4] vs 0.8 [2.5]). LVEDVi increased from 31.7 mL/m^2^ (29) to 57.7 mL/m^2^ (34) (*P* < .001), and LVSVi increased from 21 mL/m^2^ (15) to 37 mL/m^2^ (26) (*P* < .001). Indexed LV output increased, although it was not significant (2.7 L/min/m^2^ [1.7] vs 3.7 L/min/m^2^ [1.9], *P* = .093).

**Figure 1:**
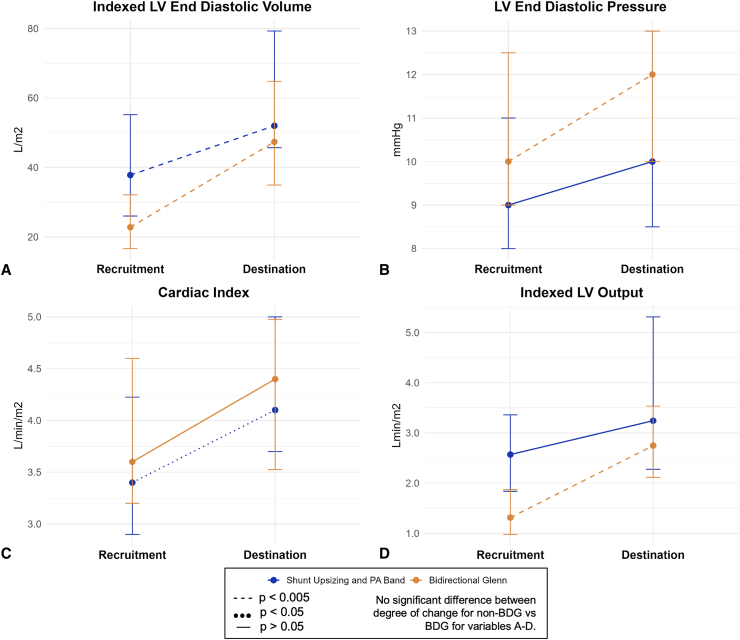
Metrics of left heart growth during staged LV recruitment. Timepoints are measured just before initial recruitment procedure and just before destination surgery (or latest follow-up). A, Indexed LV end-diastolic volume. B, LV end-diastolic pressure. C, Cardiac index (measured by Fick in catheterization laboratory). D, Indexed LV output (indexed LV stroke volume × heart rate). *BDG*, Bidirectional Glenn; *LV*, left ventricle.

Within the BDG cohort, there was no statistically significant change in MV annulus z-score from prerecruitment to destination (−2.0 [–2.3] vs −1.4 [3.0]) or AoR diameter z-score (−1.2 [0.9] vs 0.9 [1.9]). LVEDVi increased from 23 mL/m^2^ (14) to 48 mL/m^2^ (29) (*P* < .001), and LVSVi increased from 13 mL/m^2^ (9.1) to 27 mL/m^2^ (17) (*P* < .001). Indexed LV output also significantly increased from 1.7 L/min/m^2^ (1.1) to 2.9 L/min/m^2^ (1.4) (*P* < .001) (Figure 1).

**Figure 2:**
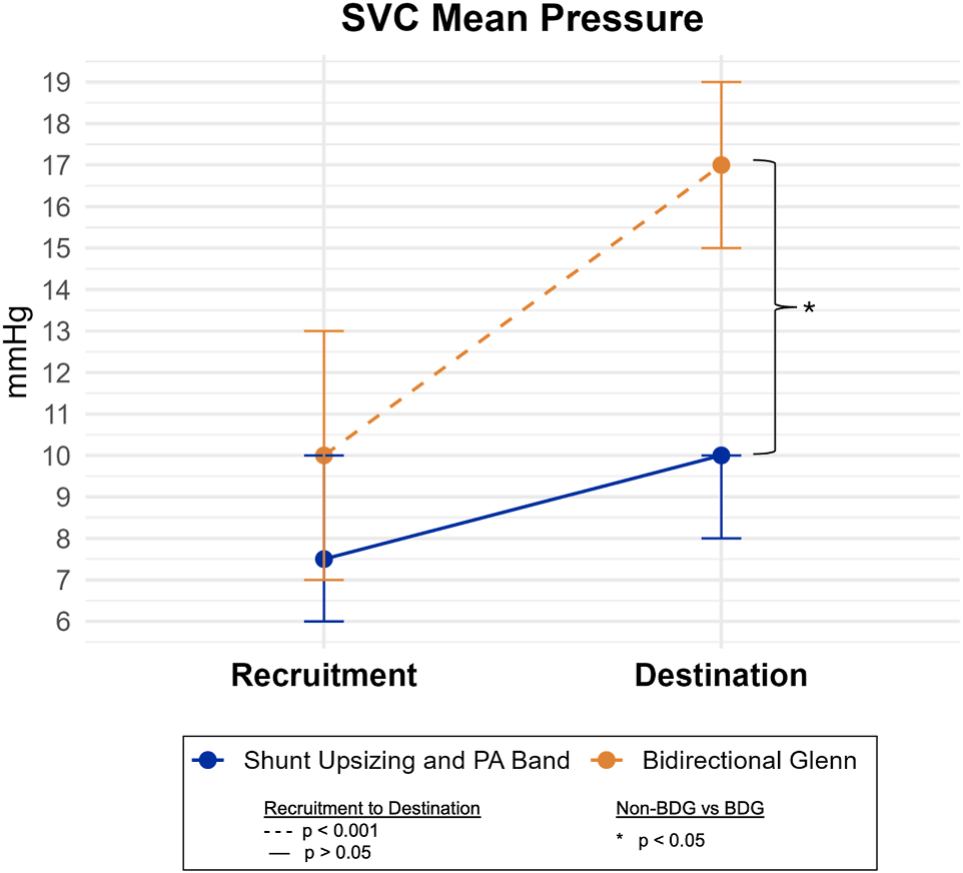
Change in mean SVC pressure from prerecruitment to predestination timepoints. Patients with a Glenn had a significant increase in SVC pressure, whereas patients undergoing shunt/Sano upsize or PAB loosening alone maintained lower SVC pressures. *SVC*, Superior vena cava; *BDG*, bidirectional Glenn; *PA*, pulmonary artery.

LVEDP went from 9.0 mm Hg (3.8) to 10.0 mm Hg (3.8) (*P* = .815) in non-BDG patients and from 10 mm Hg (3.5) to 11 mm Hg (3.8) in BDG patients (*P* = .004). Mean PA pressures for the non-BDG cohort went from 17 mm Hg (5.25) to 19 mm Hg (6) (*P* = .606) and increased significantly from 14 mm Hg (3.5) to 17.5 mm Hg (5.75) in the BDG cohort (*P* < .001). Mean SVC pressure increased from 7.5 mm Hg (4) to 10 mm Hg (2) in the non-BDG cohort (*P* = .506) and increased significantly from 10 mm Hg (6) to 17 mm Hg (4) in the BDG cohort (*P* < .001) (Figure 2). When comparing the mean change score from recruitment to destination between the cohorts, the only variable that had a significant difference in change was mean SVC pressures (*P* = .02).

### Physiologic Sequelae

There was no significant difference in the development of mild or greater mitral regurgitation at destination between non-BDG (n = 7, 53.8% of the cohort) and BDG cohorts (n = 17, 33.3%) (*P* = .29). There was a greater incidence of venovenous and aortopulmonary collateral formation and need for collateral closure in BDG patients compared with non-BDG patients (*P* < .001 for all). There were no lymphatic complications in the non-BDG cohort, whereas 16.2% of BDG patients experienced a lymphatic problem; however, this difference did not achieve statistical significance (Table 2). Lymphatic complications included 9 instances of chylothorax, 2 instances of plastic bronchitis, and no instances of protein-losing enteropathy.

**Table 2:** Outcomes

Variable	Non-BDG cohort n = 20	BDG cohort n = 68	*P* value
Lymphatic malformations diagnoses (n, %)	0 (0)	11 (16.2)	.062
Vascular sequelae			
Venovenous collateral diagnoses (n, %)	0 (0)	44 (64.7)	<.001
Venovenous collateral closure (n, %)	0 (0)	28 (41.2)	<.001
Aortopulmonary collateral diagnoses (n, %)	4 (20.0)	43 (63.2)	<.001
Aortopulmonary collateral closure (n, %)	3 (15.0)	48 (70.6)	<.001

*BDG,* Bidirectional Glenn.

## Discussion

**Figure 3:**
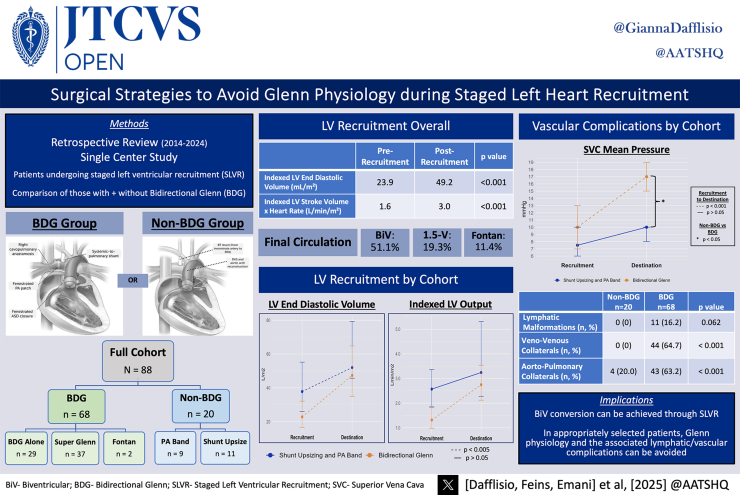
Surgical strategies to avoid Glenn physiology during staged left heart recruitment.

This study investigated the outcomes of staged LV recruitment in a cohort of patients with hypoplastic LV. Our analysis revealed that SLVR increased ventricular size and stroke volume regardless of the source of pulmonary blood flow. More than 50% of patients were able to undergo BiV conversion after SLVR. Patients with more favorable anatomy at baseline were more likely to undergo SLVR with non-BDG strategy. Patients undergoing SLVR with shunt or PAB alone demonstrated lower incidence of lymphatic malformations, venovenous collaterals, and aortopulmonary collaterals compared with SLVR with BDG or super-Glenn (Figure 3).

Several previous studies have demonstrated that successful SLVR followed by BiV conversion is possible with valve repair and volume loading of the hypoplastic ventricle.^13^^,^^14^ The super-Glenn has traditionally been our center's preference for augmenting pulmonary blood flow in the majority of patients. Recent data have demonstrated significant increases in LVEDVi from 30.0 mL/m^2^ to 52.0 mL/m^2^ and LVSVi from 16.9 mL/m^2^ to 27.6 mL/m^2^ over the recruitment period.^9^ The present study supports the existing literature by demonstrating that SLVR significantly increases LVEDVi, LVSVi, LV indexed output (LVSVi × heart rate), and cardiac index within 2 years.

Despite encouraging results of SLVR using the super-Glenn, we have observed adverse consequences of BDG physiology.^15^ At baseline for BDG, high mean SVC/Glenn pressures often leads to lymphatic complications and the development of venovenous collaterals, an effect that could be exacerbated by the addition of accessory pulmonary blood flow as part of the super-Glenn. Our center has used techniques to minimize the risk of SVC hypertension by septation within the branch PAs to separate accessory flow from the BDG. This most commonly involves placement of a 2-mm fenestrated polytetrafluoroethylene patch within the branch PAs between the Glenn and accessory pulmonary blood flow; this can also be achieved with an external branch PA band. Prasanna and colleagues^16^ demonstrated successful recruitment in patients who underwent the super-Glenn with PA septation, with a lower risk of SVC hypertension compared with the Super-Glenn alone (10% vs 44% incidence, *P* = .04). Although this technique reduces pulsatility of the SVC, it does restrict BDG flow to 1 lung, thereby increasing the resistance for the BDG circulation. In addition, LA hypertension due to SLVR may further elevate the BDG pressure.

Volume loading with shunt or native PA flow may avoid the sequelae of BDG physiology after SLVR recruitment. Although certain anatomy may predispose to PAB versus shunt placement, we consider these circulations equivalent within the non-BDG strategy, because they both provide pulsatile flow into the PA. We found that SLVR is effective in facilitating BiV conversion in 90% of patients who underwent shunt upsizing or PAB adjustment alone as the source of pulmonary blood flow. There were no significant differences in the magnitude of left heart recruitment (ie, LVEDVi, cardiac index, indexed LV output) between the 2 groups, suggesting that this is an effective strategy for SLVR.

Our analysis evaluated anatomic, volume, output, and pressure data after SLVR and found that mean SVC pressure increased significantly from pre-SLVR to post-SLVR in patients in the BDG cohort (10 mm Hg at prerecruitment to 17 mm Hg at destination), whereas the non-BDG cohort had a nonsignificant change from 7.5 to 10 mm Hg. The consequences of these elevated SVC pressures manifested with differential complication rates between the cohorts. The non-BDG cohort did not develop new lymphatic malformations or venovenous collaterals after SLVR and demonstrated a low burden of aortopulmonary collaterals. In comparison, 64.7% of the BDG cohort developed veno-venous collaterals, 63.2% developed significant APCs, and 16.2% developed lymphatic malformations. When looking specifically at patients most susceptible to lymphatic complications, no patient with HLHS or a stage 1 Norwood developed lymphatic malformations in the non-BDG cohort. In comparison, 16.2% (n = 6/37) of HLHS patients in the BDG cohort and 14.5% (n = 6/55) of stage 1 Norwood patients in the BDG cohort did.

**Figure 4:**
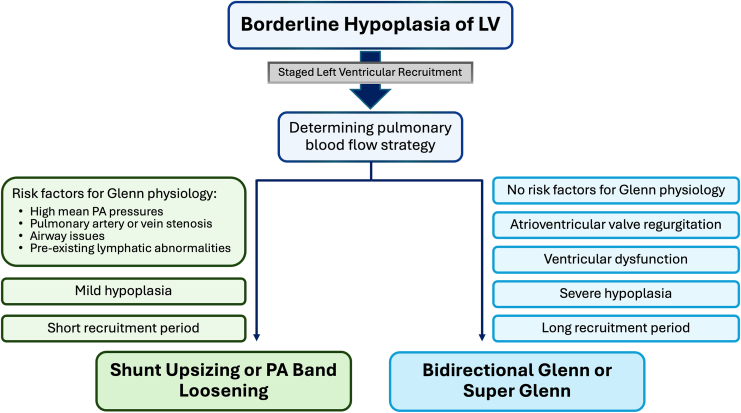
Algorithm for determining pulmonary blood flow strategy during staged LV recruitment of borderline left heart hypoplasia. Shunt upsizing or PA band loosening is preferable in patients with risk factors for Glenn physiology or those who have mild hypoplasia and a predicted short recruitment period. *LV*, Left ventricle; *PA*, pulmonary artery.

The choice of pulmonary blood flow in patients undergoing SLVR requires careful consideration (Figure 4). In patients with risk factors for BDG, such as high mean PA pressures, PA or vein stenosis, or preexisting lymphatic abnormalities, BDG or super-Glenn physiology may put the patient at a high risk for SVC hypertension or venovenous collaterals. In this patient population, we would prefer shunt upsizing or PAB loosening. For patients who demonstrate mild degrees of hypoplasia, a short period of recruitment may be sufficient and shunt strategy may be preferable. A BDG strategy may be preferred for patients without risk factors for BDG physiology. Moreover, shunt physiology may be detrimental in certain patients with atrioventricular valve regurgitation or ventricular dysfunction. Finally, patients with severe hypoplasia may require a long duration of SLVR and most likely require Fontan or a nonanatomic 1.5-ventricle repair as ultimate circulation. In these patients, BDG may be a suitable strategy to gently load the LV while keeping options open for ultimate SVP or reverse 1.5 circulation. For patients in whom the anticipated time to ventricular growth may be prolonged, BDG may provide a more stable degree of pulmonary blood flow compared with a shunt or PAB (Video 1).

In patients undergoing shunt/PAB, it is important to calibrate the degree of pulmonary blood flow carefully at the time of SLVR to avoid excessive pulmonary blood flow. Patients must be monitored carefully to ensure adequate flow with somatic growth. At the time of SLVR, distal PA pressures and estimates of Qp:QS are assessed in the operating room. We aim for a Qp:Qs of 1.5 to 1.0 with even distribution of blood flow to both lungs. Distal PA pressures are assessed to ensure we are not undertreating distal PA hypertension, and the goal mean PA pressure is less than 20 mm Hg. If distal PA pressure is high, we investigate whether it is caused by pulmonary vascular resistance or elevated LA pressure. If the cause is LA pressure, we may further open the atrial septum. If the cause is pulmonary vascular resistance, we would pursue vasodilation and may start nitric oxide in the operating room to understand what would happen if we committed to a shunt size. Although the goal of augmenting flow is to volume load the LV, excessive pulmonary blood flow can predispose to pulmonary and left atrial hypertension, which may disrupt candidacy for SVP. In some cases, an upsized shunt will need to be clipped/restricted slightly in the operating room to ensure acceptable PA pressures with the ability to subsequently dilate the shunt in the catheterization laboratory. In patients with dual sources of pulmonary blood flow, magnetic resonance imaging may be the best method to assess flow in the interstage.

### Limitations

This study has limitations due to its nature as a retrospective review. In addition, the small numbers of the non-BDG group made meaningful paired comparison difficult, and it is possible that this analysis misses significant differences between the 2 groups. Because of the small non-BDG cohort, we were also unable to match cohorts, although a propensity-matched analysis evaluating the efficacy of shunt/Sano upsizing and PAB would be beneficial. Because of small numbers, we were also unable to compare patients within the non-BDG cohort who may have gone down this pathway for different reasons. Some avoided a BDG due to less severe left heart hypoplasia, whereas others did not undergo a BDG because they were poor Glenn candidates.

## Conclusions

Shunt/Sano upsizing and PA band modulation can be effective sources of pulmonary blood flow augmentation to increase LV volume loading and output during staged LV recruitment. Successful BiV conversion can be achieved while avoiding BDG physiology and associated lymphatic/vascular sequelae. This may be an effective recruitment strategy for patients with less severe disease at baseline or those who cannot tolerate BDG physiology.

### Webcast

You can watch a Webcast of this AATS meeting presentation by going to: https://www.aats.org/resources/shunt-upsizing-as-a-strategy-t-9779.

**Figure:**
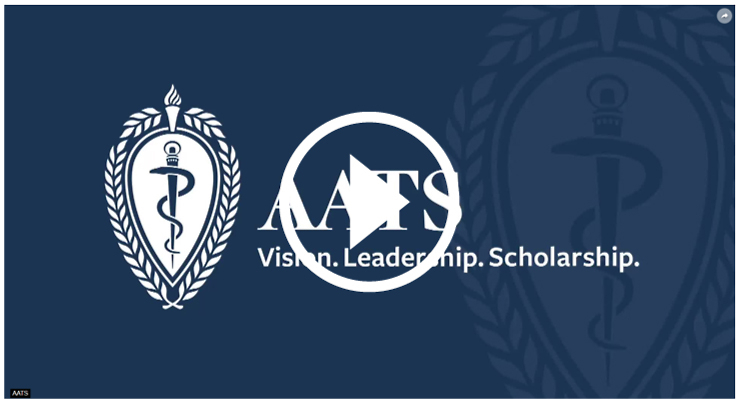

### Audio

You can listen to the discussion audio of this article by going to the supplementary material section below.

## Conflict of Interest Statement

The authors reported no conflicts of interest.

The *Journal* policy requires editors and reviewers to disclose conflicts of interest and to decline handling or reviewing manuscripts for which they may have a conflict of interest. The editors and reviewers of this article have no conflicts of interest.

## Supplementary Data

### Audio

Audio 1 This is an audio recording of the presentation of the associated abstract and discussion. The abstract presentation ends at 7:10 and the discussion begins at 7:16.

Video 1 Overview of strategy for shunt upsizing in patients undergoing staged ventricular recruitment. Video available at: https://www.jtcvs.org/article/S2666-2736(26)00347-5/fulltext.

Video 2 Video available at: https://www.jtcvs.org/article/S2666-2736(26)00347-5/fulltext.
